# l-Leucinium fluoride monohydrate

**DOI:** 10.1107/S1600536812039001

**Published:** 2012-09-19

**Authors:** Ouahida Zeghouan, Lamia Bendjeddou, Aouatef Cherouana, Slimane Dahaoui, Claude Lecomte

**Affiliations:** a Unité de Recherche Chimie de l’Environnement et Molculaire Structurale (CHEMS), Faculté des Sciences Exactes, Campus Chaabet Ersas, Université Mentouri de Constantine, 25000 Constantine, Algeria; b Cristallographie, Résonance Magnétique et Modélisation (CRM2), Université Henri Poincaré, Nancy 1, Faculté des Sciences, BP 70239, 54506 Vandoeuvre lès Nancy CEDEX, France

## Abstract

The asymmetric unit of the title hydrated salt, C_6_H_14_NO_2_^+^·F^−^·H_2_O, contains a discrete cation with a protonated amino group, a halide anion and one water mol­ecule. The crystal structure is composed of double layers parallel to (010) held together by N—H⋯O, N—H⋯F, O—H⋯F and C—H⋯F hydrogen bonds, forming a two-dimensional network, and stacked along the *c* axis, *viz*. hydro­philic layers at *z* = 0 and 1/2 and hydro­phobic layers at *z* = 1/3 and 2/3.

## Related literature

For hydrogen-bond motifs, see: Bernstein *et al.* (1995 ▶). For background to carb­oxy­lic acids, see: Miller & Orgel (1974 ▶); Kvenvolden *et al.* (1971 ▶). For our research on organic salts of amino acids, see: Guenifa *et al.* (2009 ▶); Moussa Slimane *et al.* (2009 ▶). For l-leucinium oxalate, see: Rajagopal *et al.* (2003 ▶) and for l-leucinium perchlorate, see: Janczak & Perpétuo (2007 ▶).
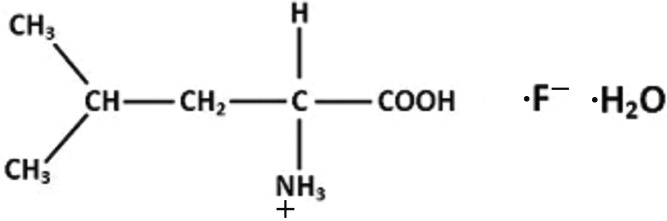


## Experimental

### 

#### Crystal data

C_6_H_14_NO_2_^+^·F^−^·H_2_O
*M* *_r_* = 169.20
Orthorhombic, 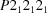

*a* = 5.7058 (1) Å
*b* = 5.8289 (1) Å
*c* = 27.3150 (4) Å
*V* = 908.46 (3) Å^3^
*Z* = 4
Mo *K*α radiation
μ = 0.11 mm^−1^
*T* = 100 K
0.3 × 0.03 × 0.02 mm

#### Data collection

Oxford Diffraction Super Nova diffractometer with an Atlas detector
27972 measured reflections
2771 independent reflections
2584 reflections with *I* > 2σ(*I*)
*R*_int_ = 0.039

#### Refinement

*R*[*F* ^2^ > 2σ(*F* ^2^)] = 0.030
*wR*(*F* ^2^) = 0.078
*S* = 1.06
2771 reflections
118 parameters
7 restraints
H atoms treated by a mixture of independent and constrained refinement
Δρ_max_ = 0.28 e Å^−3^
Δρ_min_ = −0.14 e Å^−3^

### 

Data collection: *CrysAlis CCD* (Oxford Diffraction, 2008 ▶); cell refinement: *CrysAlis CCD*; data reduction: *CrysAlis RED* (Oxford Diffraction, 2008 ▶); program(s) used to solve structure: *SIR92* (Altomare *et al.*, 1993 ▶); program(s) used to refine structure: *SHELXL97* (Sheldrick, 2008 ▶); molecular graphics: *ORTEPIII* (Farrugia, 1997 ▶); software used to prepare material for publication: *WinGX* (Farrugia, 1999 ▶), *PARST97* (Nardelli, 1995 ▶), *Mercury* (Macrae *et al.*, 2006 ▶) and *POVRay* (Persistence of Vision Team, 2004 ▶).

## Supplementary Material

Crystal structure: contains datablock(s) global, I. DOI: 10.1107/S1600536812039001/aa2065sup1.cif

Structure factors: contains datablock(s) I. DOI: 10.1107/S1600536812039001/aa2065Isup2.hkl

Supplementary material file. DOI: 10.1107/S1600536812039001/aa2065Isup3.cml

Additional supplementary materials:  crystallographic information; 3D view; checkCIF report

## Figures and Tables

**Table 1:** Hydrogen-bond geometry (Å, °)

*D*—H⋯*A*	*D*—H	H⋯*A*	*D*⋯*A*	*D*—H⋯*A*
N1—H1*N*⋯O1*W* ^i^	0.91 (2)	1.94 (2)	2.8428 (11)	174 (2)
N1—H2*N*⋯F1^ii^	0.879 (17)	1.878 (17)	2.7277 (10)	162.1 (16)
N1—H3*N*⋯O1*W* ^iii^	0.89 (2)	1.95 (2)	2.8152 (11)	166 (2)
O1—H1⋯F1^iv^	0.88 (2)	1.57 (2)	2.4410 (10)	174 (2)
O1*W*—H1*W*⋯F1	0.84 (1)	1.87 (1)	2.7090 (9)	174 (1)
O1*W*—H2*W*⋯F1^ii^	0.83 (1)	1.90 (1)	2.7271 (9)	170 (1)
C4—H4⋯F1^ii^	0.98	2.45	3.3813 (12)	159

Symmetry codes: (i) 
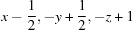
; (ii) 
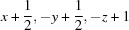
; (iii) 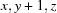; (iv) 
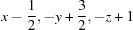
.

**Fig. 1.**
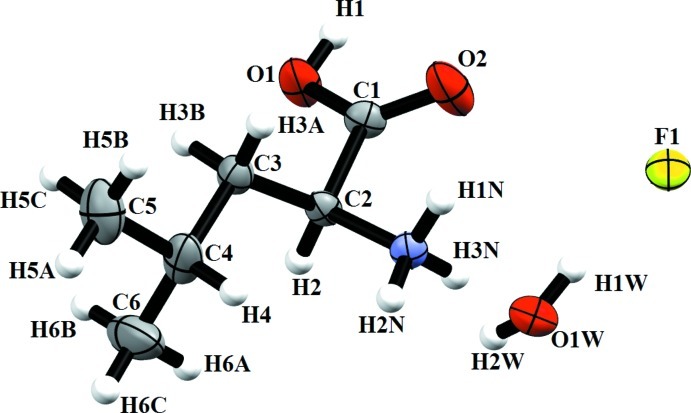
The asymmetric unit of the title compound, showing the crystallographic numbering scheme. Displacement ellipsoids are drawn at the 50% probability level and H atoms are shown as spheres of arbitrary radii.

**Fig. 2.**
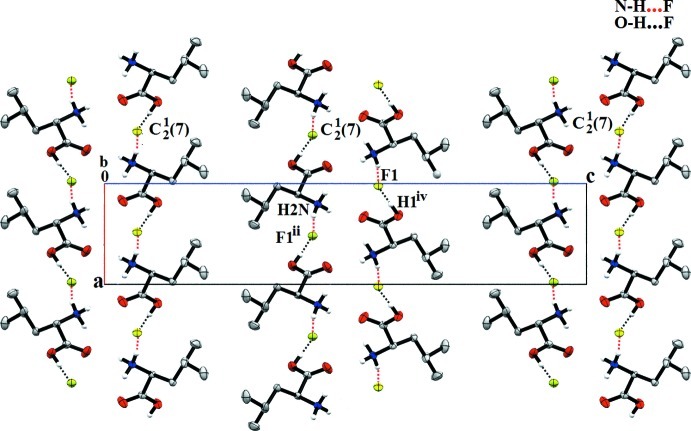
Part of the crystal structure, showing the aggregation of C12(7) for the title compound. [Symmetry codes: (ii) x + 1/2, -y + 1/2, -z + 1; (iv) x - 1/2, -y + 3/2, -z + 1]. For the sake of clarity, the water molecules and H atoms not involved in hydrogen bonding have been omitted.

**Fig. 3.**
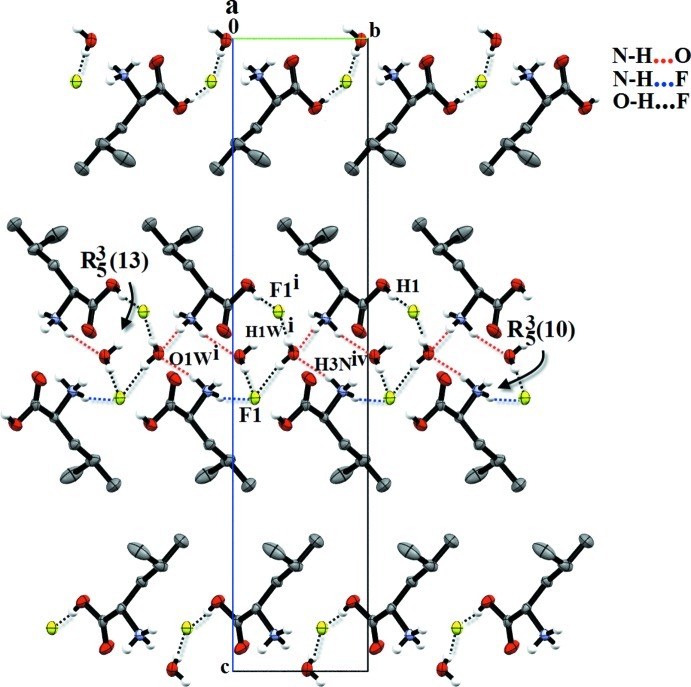
Packing view of the title compound showing the aggregation of R35(10) and R35(15) hydrogen-bonding motifs. [Symmetry codes:(i) x - 1/2, -y + 1/2, -z + 1; (iv) x - 1/2, -y + 3/2, -z + 1].

## supplementary crystallographic information

### Comment

Leucine is one of the most important amino acids, essential for the growth and maintenance of living organisms. Simple carboxylic acids, which are believed to have existed in the prebiotic earth (Miller & Orgel, 1974; Kvenvolden *et al.*, 1971), form crystalline complexes with amino acids. The present paper is a part of our research with organic salts of amino acids (Guenifa *et al.*, 2009; Moussa Slimane *et al.*, 2009).

The asymmetric unit of the title compound contains a leucinium cation, fluoride anion and one water molecule (Fig. 1). As expected, leucine form the protonated unit with the transfer of an H atom from the inorganic acid. The similar situation is observed in *L*-leucinium oxalate (Rajagopal *et al.*, 2003) and *L*-leucinium perchlorate (Janczak & Perpétuo, 2007).

In the supramolecular structure of the title compound, the ions are connected into a two-dimensional hydrogen-bonded network *via* N—H···O, N—H···F, O—H···F and C—H···F hydrogen bonds (Table 1). The leucinium cations are interlinked by two intermolecular N—H···F and O—H···F hydrogen bonds to form a double layers [C^1^_2_(7) motif] (Bernstein *et al.*,, 1995), (Fig. 2), resulting in an overall one-dimensional hydrogen-bonded network.

In the title compound, the water molecules and floride anions bridges in two-dimensional hydrogen bonded network, forming a non centrosymmetric hydrogen-bonded *R*^3^_5_(13) and *R*^3^_5_(10) motifs, which run into zigzag parallel to the [010] direction (Fig. 3).

The molecular packing of the title compound consists of double layers is stacked along the *c* axis, *viz*. hydrophilic layers at *z* = 0 and 1/2 and hydrophobic layers at *z* = 1/3 and 2/3. The hydrophilic layers include the head of the leucinium residue (ammonium and carboxylic groups), floride anion and water molecule.

### Experimental

The experiment consists of heating an equimolar solution of leucine and hydrofluoric acid acid until the reaction is complete. Colourless crystal with melting points of 618 K were obtained by evaporation of the solution at room temperature over the course of a few days.

### Refinement

The H atoms attached to C atoms were placed at calculated positions with C—H fixed at 0.93 – 0.98 Å The H atoms attached to N and O were initially located from difference maps and refined with distance restraint for the N—H bond length 0.90 (2) Å and O—H bond length 0.85 (2) Å. The U_iso_(H) were set to 1.5U_eq_(C, O) for methyl and amino groups and to 1.2U_eq_(C, N) for the rest atoms.

### Crystal data

**Table:**

C_6_H_14_NO_2_^+^·F^−^·H_2_O	*F*(000) = 368
*M**_r_* = 169.20	*D*_x_ = 1.237 Mg m^−^^3^
Orthorhombic, *P*2_1_2_1_2_1_	Mo *K*α radiation, λ = 0.71073 Å
Hall symbol: P 2ac 2ab	Cell parameters from 27972 reflections
*a* = 5.7058 (1) Å	θ = 3.6–30.5°
*b* = 5.8289 (1) Å	µ = 0.11 mm^−^^1^
*c* = 27.3150 (4) Å	*T* = 100 K
*V* = 908.46 (3) Å^3^	Needle, colourless
*Z* = 4	0.3 × 0.03 × 0.02 mm

### Data collection

**Table:**

Oxford Diffraction Super Nova diffractometer with an Atlas detector	2584 reflections with *I* > 2σ(*I*)
Radiation source: Enhance (Mo) X-ray Source	*R*_int_ = 0.039
Graphite monochromator	θ_max_ = 30.5°, θ_min_ = 3.6°
Detector resolution: 10.4508 pixels mm^-1^	*h* = −8→8
ω scans	*k* = −8→8
27972 measured reflections	*l* = −39→39
2771 independent reflections	

### Refinement

**Table:**

Refinement on *F*^2^	7 restraints
Least-squares matrix: full	H atoms treated by a mixture of independent and constrained refinement
*R*[*F*^2^ > 2σ(*F*^2^)] = 0.030	*w* = 1/[σ^2^(*F*_o_^2^) + (0.045*P*)^2^ + 0.0976*P*] where *P* = (*F*_o_^2^ + 2*F*_c_^2^)/3
*wR*(*F*^2^) = 0.078	(Δ/σ)_max_ = 0.001
*S* = 1.06	Δρ_max_ = 0.28 e Å^−^^3^
2771 reflections	Δρ_min_ = −0.14 e Å^−^^3^
118 parameters	

### Special details

**Table:**

Geometry. All e.s.d.'s (except the e.s.d. in the dihedral angle between two l.s. planes) are estimated using the full covariance matrix. The cell e.s.d.'s are taken into account individually in the estimation of e.s.d.'s in distances, angles and torsion angles; correlations between e.s.d.'s in cell parameters are only used when they are defined by crystal symmetry. An approximate (isotropic) treatment of cell e.s.d.'s is used for estimating e.s.d.'s involving l.s. planes.
Refinement. Refinement of *F*^2^ against ALL reflections. The weighted *R*-factor *wR* and goodness of fit *S* are based on *F*^2^, conventional *R*-factors *R* are based on *F*, with *F* set to zero for negative *F*^2^. The threshold expression of *F*^2^ > σ(*F*^2^) is used only for calculating *R*-factors(gt) *etc*. and is not relevant to the choice of reflections for refinement. *R*-factors based on *F*^2^ are statistically about twice as large as those based on *F*, and *R*- factors based on ALL data will be even larger.

### Fractional atomic coordinates and isotropic or equivalent isotropic displacement parameters (Å^2^)

**Table:**

	*x*	*y*	*z*	*U*_iso_*/*U*_eq_	
F1	0.01929 (10)	0.16104 (10)	0.56810 (2)	0.01618 (13)	
O1	−0.18388 (14)	1.11050 (12)	0.38989 (3)	0.01957 (16)	
O1W	0.35872 (12)	0.05014 (12)	0.50292 (3)	0.01595 (15)	
H1W	0.248 (2)	0.090 (2)	0.5214 (4)	0.024*	
H2W	0.404 (2)	0.152 (2)	0.4837 (4)	0.024*	
O2	−0.16963 (15)	0.93446 (14)	0.46240 (3)	0.02415 (18)	
N1	0.20572 (15)	0.68869 (14)	0.44249 (3)	0.01236 (15)	
C2	0.10845 (16)	0.82520 (16)	0.40113 (3)	0.01163 (16)	
H2	0.2288	0.9319	0.3894	0.014*	
C3	0.02982 (17)	0.67306 (17)	0.35844 (3)	0.01419 (17)	
H3B	−0.0459	0.7694	0.3342	0.017*	
H3A	−0.0867	0.5658	0.3705	0.017*	
C1	−0.09782 (17)	0.96280 (16)	0.42137 (3)	0.01297 (17)	
C4	0.2246 (2)	0.5363 (2)	0.33310 (4)	0.0212 (2)	
H4	0.3018	0.4393	0.3576	0.025*	
C5	0.1160 (2)	0.38189 (19)	0.29402 (4)	0.0269 (2)	
H5A	0.2373	0.296	0.278	0.04*	
H5C	0.0351	0.4746	0.2704	0.04*	
H5B	0.0072	0.278	0.3091	0.04*	
C6	0.4082 (2)	0.6941 (3)	0.31019 (5)	0.0349 (3)	
H6A	0.4746	0.7906	0.3351	0.052*	
H6B	0.3356	0.7877	0.2856	0.052*	
H6C	0.5297	0.6032	0.2955	0.052*	
H1	−0.294 (3)	1.184 (3)	0.4060 (7)	0.052*	
H2N	0.317 (3)	0.593 (3)	0.4337 (6)	0.042*	
H1N	0.090 (3)	0.610 (3)	0.4578 (6)	0.042*	
H3N	0.263 (3)	0.784 (3)	0.4649 (5)	0.042*	

### Atomic displacement parameters (Å^2^)

**Table:**

	*U*^11^	*U*^22^	*U*^33^	*U*^12^	*U*^13^	*U*^23^
F1	0.0160 (3)	0.0144 (3)	0.0181 (3)	−0.0065 (2)	0.0001 (2)	0.0000 (2)
O1	0.0231 (4)	0.0191 (3)	0.0164 (3)	0.0124 (3)	0.0043 (3)	0.0044 (3)
O1W	0.0147 (3)	0.0115 (3)	0.0216 (3)	0.0002 (3)	0.0031 (3)	0.0007 (3)
O2	0.0285 (4)	0.0232 (4)	0.0207 (4)	0.0140 (3)	0.0102 (3)	0.0085 (3)
N1	0.0130 (4)	0.0112 (3)	0.0129 (3)	0.0033 (3)	0.0003 (3)	0.0004 (3)
C2	0.0121 (4)	0.0102 (3)	0.0126 (4)	0.0032 (3)	0.0014 (3)	0.0015 (3)
C3	0.0152 (4)	0.0143 (4)	0.0130 (4)	0.0026 (4)	−0.0006 (3)	−0.0015 (3)
C1	0.0136 (4)	0.0093 (4)	0.0160 (4)	0.0023 (3)	0.0007 (3)	−0.0001 (3)
C4	0.0251 (5)	0.0235 (5)	0.0149 (4)	0.0123 (4)	−0.0013 (4)	−0.0040 (4)
C5	0.0398 (6)	0.0215 (5)	0.0193 (5)	0.0034 (5)	0.0028 (5)	−0.0061 (4)
C6	0.0188 (5)	0.0536 (8)	0.0322 (6)	−0.0022 (5)	0.0076 (5)	−0.0192 (6)

### Geometric parameters (Å, º)

**Table:**

O1—C1	1.3121 (12)	C4—C5	1.5276 (16)
O2—C1	1.2047 (12)	C4—C6	1.5281 (18)
O1—H1	0.879 (18)	C2—H2	0.9800
O1W—H1W	0.841 (11)	C3—H3B	0.9700
O1W—H2W	0.834 (11)	C3—H3A	0.9700
N1—C2	1.4891 (12)	C4—H4	0.9800
N1—H2N	0.879 (17)	C5—H5B	0.9600
N1—H3N	0.889 (16)	C5—H5C	0.9600
N1—H1N	0.906 (17)	C5—H5A	0.9600
C1—C2	1.5278 (13)	C6—H6C	0.9600
C2—C3	1.5321 (12)	C6—H6A	0.9600
C3—C4	1.5329 (15)	C6—H6B	0.9600
			
C1—O1—H1	105.0 (12)	C2—C3—H3A	108.00
H1W—O1W—H2W	114.5 (11)	C2—C3—H3B	108.00
H2N—N1—H3N	108.6 (16)	C4—C3—H3B	108.00
H1N—N1—H3N	105.5 (15)	H3A—C3—H3B	107.00
C2—N1—H1N	110.4 (11)	C4—C3—H3A	108.00
C2—N1—H2N	113.7 (11)	C5—C4—H4	109.00
C2—N1—H3N	109.0 (10)	C6—C4—H4	109.00
H1N—N1—H2N	109.4 (16)	C3—C4—H4	109.00
O1—C1—O2	124.92 (9)	C4—C5—H5A	109.00
O2—C1—C2	121.78 (8)	C4—C5—H5B	110.00
O1—C1—C2	113.30 (7)	H5A—C5—H5B	109.00
N1—C2—C3	112.16 (8)	H5A—C5—H5C	110.00
C1—C2—C3	110.71 (7)	C4—C5—H5C	109.00
N1—C2—C1	107.04 (7)	H5B—C5—H5C	109.00
C2—C3—C4	115.62 (8)	C4—C6—H6B	109.00
C3—C4—C5	109.14 (9)	C4—C6—H6C	110.00
C3—C4—C6	111.65 (10)	C4—C6—H6A	109.00
C5—C4—C6	110.28 (10)	H6A—C6—H6C	110.00
N1—C2—H2	109.00	H6B—C6—H6C	109.00
C3—C2—H2	109.00	H6A—C6—H6B	109.00
C1—C2—H2	109.00		
			
O1—C1—C2—N1	172.87 (8)	N1—C2—C3—C4	−63.80 (10)
O1—C1—C2—C3	−64.60 (10)	C1—C2—C3—C4	176.70 (8)
O2—C1—C2—N1	−6.99 (12)	C2—C3—C4—C5	176.10 (8)
O2—C1—C2—C3	115.54 (10)	C2—C3—C4—C6	−61.73 (11)

### Hydrogen-bond geometry (Å, º)

**Table:**

*D*—H···*A*	*D*—H	H···*A*	*D*···*A*	*D*—H···*A*
N1—H1*N*···O1*W*^i^	0.91 (2)	1.94 (2)	2.8428 (11)	174 (2)
N1—H2*N*···F1^ii^	0.879 (17)	1.878 (17)	2.7277 (10)	162.1 (16)
N1—H3*N*···O1*W*^iii^	0.89 (2)	1.95 (2)	2.8152 (11)	166 (2)
O1—H1···F1^iv^	0.88 (2)	1.57 (2)	2.4410 (10)	174 (2)
O1*W*—H1*W*···F1	0.84 (1)	1.87 (1)	2.7090 (9)	174 (1)
O1*W*—H2*W*···F1^ii^	0.83 (1)	1.90 (1)	2.7271 (9)	170 (1)
C4—H4···F1^ii^	0.98	2.45	3.3813 (12)	159

Symmetry codes: (i) *x*−1/2, −*y*+1/2, −*z*+1; (ii) *x*+1/2, −*y*+1/2, −*z*+1; (iii) *x*, *y*+1, *z*; (iv) *x*−1/2, −*y*+3/2, −*z*+1.
